# Analysis of metadynamics simulations by metadynminer.py

**DOI:** 10.1093/bioinformatics/btae614

**Published:** 2024-10-18

**Authors:** Jan Beránek, Aleš Křenek, Vojtěch Spiwok

**Affiliations:** Department of Biochemistry and Microbiology, University of Chemistry and Technology, Prague, Prague 6 166 28, Czech Republic; Institute of Computer Science, Masaryk University, Brno 602 00, Czech Republic; Department of Biochemistry and Microbiology, University of Chemistry and Technology, Prague, Prague 6 166 28, Czech Republic

## Abstract

**Motivation:**

Molecular dynamics simulation is very useful but computationally demanding method of studying dynamics of biomolecular systems. Many enhanced sampling methods were developed in order to obtain the desired results in available computational time. Metadynamics and its variants are common enhanced sampling methods used for this purpose. Metadynamics simulations allow the user to gather large amounts of data, which have to be analyzed to elucidate the properties of the studied system.

**Results:**

Here, we present metadynminer.py, a Python package that allows easy and user-friendly analysis and visualization of the results obtained from metadynamics simulations. The built-in functions automate frequent tasks and make the package easy to use for new users, while its many customization options and object-oriented nature allow for integration into specialized data analysis workflows by more advanced users.

**Availability and implementation:**

The “metadynminer.py” Python package is available under the GPL-3.0 license via PyPi and Conda. The development version is available on GitHub along with issue support (https://github.com/Jan8be/metadynminer.py). Documentation, tutorial and Jupyter Notebook (provided through the public mybinder.org service) are available at https://metadynreporter.cz.

## Graphical abstract

**Graphical Abstract:**
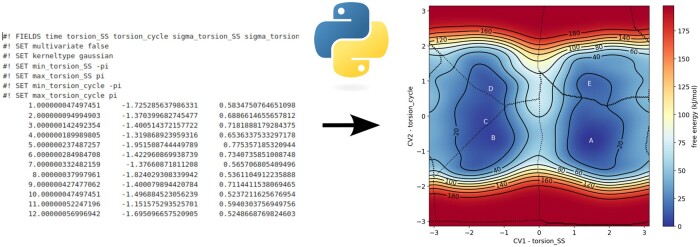

## 1 Introduction 

To elucidate the function of biomolecular machines and control their functions, it is necessary to understand not only structure but also dynamics. Dynamics of molecular systems can be expressed in terms of populations of different states, such as conformations of proteins and nucleic acids or bound/unbound states of molecular complexes. Observation of a molecular system for a long period of time allows one to determine populations of these states. Molecular dynamics simulation is one of the methods that makes such observations possible using a computer. This makes the method an attractive tool for ligand design and for studying the stability of biomolecules, among other applications.

Molecular dynamics simulation is a computationally expensive method. This limits accessible time scales to microseconds in atomistic simulations of biomolecular systems. Therefore, slow and rarely occurring processes, such as protein folding or ligand (un)binding, are extremely difficult to study. Metadynamics (Laio and Parrinello 2002) is one of the methods developed to address this problem. This method makes it possible to study long time scales from relatively short simulations. Since its introduction in 2002, it has been applied in thousands of simulations, for example, in drug discovery, to trace allosteric motions in proteins, in protein misfolding and aggregation, and other fields (Comitani and Gervasio 2018), also outside the field of biomolecular systems.

Metadynamics accelerates the simulation by disfavoring previously visited states of the system. This is done using an artificial bias potential *V*. The bias potential is a function of a few (typically one–three, in most cases two) descriptors of the state of the system, called collective variables (**s**), selected prior to the simulation. In the simulation, small Gaussian-shaped potentials that are function of collective variables (“hills”) accumulate during the simulation to form the bias potential, which “floods” already explored states.

The most stable states of the system tend to be sampled for the longest time, and thus the highest bias potential is required to disfavor them. Therefore, the bias potential can be used as a measure of stability. Thermodynamically speaking, the free energy surface *F* is proportional to a negative image of the bias potential (F(s)∝−V(s)). Free energy differences can be used to predict the equilibrium constants of the processes studied.

To make efficient calculation, visualization, and analysis of free energy surfaces possible, we developed a Web-based tool Metadynview (Hošek and Spiwok 2016) and the R packages Metadynminer and Metadynminer3D (Trapl and Spiwok 2022). In this article, we present the next generation of the software extended with new functionalities. Due to the increasing popularity of the Python programming language, it has also been reimplemented in Python natively. A typical (yet extensible) workflow is provided as a Jupyter notebook, which can be run on the public mybinder.org service in a single click.

## 2 Software functions

The library can be used to analyze results from metadynamics (Laio and Parrinello 2002), including well-tempered (Barducci *et al.* 2008) or parallel tempering metadynamics (Bussi *et al.* 2006). The library is primarily intended to be used with Plumed software (Tribello *et al.* 2014), which is a well-established program that is used to introduce metadynamics and other accelerated sampling methodologies to the most popular simulation software. A Plumed output file contains the list of hills with their heights and widths. Since this is a text-based format, input files from metadynamics engines other than Plumed can be analyzed by metadynminer.py after a simple format conversion.

### 2.1 Calculation of free energy surfaces

There are two ways to predict the free energy surfaces from metadynamics simulations, from the bias potential and from a combination of the bias potential and populations of the states. The former, faster and less accurate method, is natively implemented in metadynminer.py. The latter, slower and more accurate method, can also be implemented due to the flexibility of the metadynminer.py library (tutorial provided).

Calculation of the free energy surface from the bias potential represents the summation of Gaussian hills forming the bias potential. Next, the negative value is provided as the estimate of the free energy surface (F(s)=−V(s)). For well-tempered metadynamics (which is currently the most popular variant), the negative value of the bias potential must be scaled (F(s)=−αV(s)). Plumed pre-scales heights of hills, so no action is required from the user.

The summation of hills can be a non-trivial task, because hills are usually being deposited every few picoseconds, thus the bias potential from the whole simulation can be formed by tens of thousands or more hills. For this purpose, we use our Hillsum algorithm (Hošek and Spiwok 2016) (function Fes with keyword original=False). This algorithm uses a precomputed Gaussian hill to replace computationally expensive evaluations of the Gaussian function. The function is fast and the output is reasonably accurate. It is ideal for visualization purposes.

The function Fes with keyword original=True explicitly evaluates the Gaussian function for each hill. It is more precise and gives the same result as Plumed’s sum_hills (see Supplementary Data for comparisons); therefore, it is suitable for quantitative predictions.

The library is written in an object-oriented style. Therefore, hills, free energy surfaces, and other data are stored as objects, and users can apply specific functions to them or access their attributes for further analysis or visualization purposes. It is possible to sum or subtract two free energy surfaces (e.g., for a comparison of two systems) or multiply it by a scalar value (e.g., to convert the free energy surface unit from kJ/mol to kcal/mol).

### 2.2 Visualization

**Figure 1.**
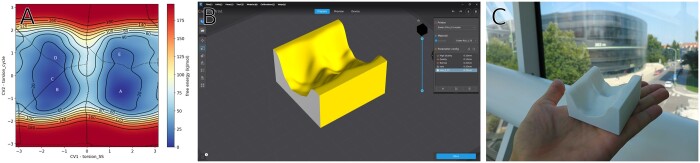
Example of free energy surface visualization made with metadynminer.py. (A) Free energy surface plot. Letters in the picture depict the positions of local free energy minima, solid contours depict selected free energy isovalues, dotted lines depict boundaries between local minima as detected by algorithm introduced in metadynminer.py. (B) stl file produced with the metadynminer.py and numpy-stl libraries, viewed in slicing software. (C) Resulting 3D print of the stl file

Metadynminer.py uses a popular Matplotlib library (Hunter 2007) to plot free energy surfaces with one or two collective variables. PyVista 3D visualization library (Sullivan and Kaszynski 2019) is used for free energy surfaces with three collective variables. Users can customize plots by adjusting the axis range, changing a color palette, etc. An example of a two-dimensional conformational free energy surface of oxytocin from metadynminer.py is depicted in Fig. 1. Associated data are presented in Plumed Nest (The PLUMED consortium 2019) and Supplementary Data. The labels of the axes are created automatically from the depiction of the HILLS file, or they can be supplied by the user. The letters in the local minima change the color automatically for optimal readability depending on the background, but again the color can be overridden by user settings. Figures can be exported in publication quality.

### 2.3 Further analyses

There are other functionalities of metadynminer.py. It has built-in functions to plot how the values of collective variables change during simulation. It can also plot the heights of the hills in well-tempered metadynamics (Barducci *et al.* 2008).

The library has function Minima which, when used with keyword precise=True, automatically detects local free energy minima, which correspond to metastable states. Boundaries between metastable states, in other words free energy barriers, between local minima are automatically detected. These boundaries are also visible in Fig. 1A. For each point on the free energy surface belonging to some minimum, its relative population is predicted and the probabilities are summed. The sums of probabilities for each minima are converted back to free energies. In this way, the value of the free energy of the minima accounts for both the depths and the widths of the free energy minima.

Sometimes, it is not useful to localize all local minima of some rough free energy surface. When using the function Minima with the keyword precise=False, metadynminer.py divides the range of collective variables into a grid (by default 8, 8 × 8 or 8 × 8 × 8 for 1D, 2D, or 3D free energy surface, respectively). For each cell of the grid, the library finds its lowest point and, if it proves to be a local minimum, adds it to the list of local minima. Therefore, it can find up to 64 minima of a 2D free energy surface in the default settings.

Furthermore, it is possible to calculate and plot the predicted free energies for all minima along the simulation (relative to the global minimum). These profiles could be used as an indication of convergence of the simulation toward stable free energy predictions (it must be kept in mind that the stability of free energy predictions is one of, not the only indicators of convergence).

Another way to visualize the convergence of simulation is the removal of one of the collective variables (e.g. conversion of a 2D free energy surface to 1D). This can be plotted and compared for different stages of the simulation. This option is also supported by metadynminer.py.

One of the reasons why we have decided to implement this library in the Python language is that it allows easy integration of metadynminer.py with other Python-based libraries commonly used for visualization or data analysis. Examples of such integration are:

- Exporting free energy surface plots as html web objects using the mpld3 library (Vanderplas 2014) (or similar).
- Loading free energy surface calculated by metadynminer.py to numpy-stl library, to export it as an stl file which can be 3D printed for presentation purposes (Fig. 1B and C).
- Estimation of the errors of the predicted free energy differences by autocorrelation analysis. Reweighted populations of minima can be analyzed using fast numeric libraries to sample uncorrelated samples (Klimovich *et al.* 2015). These can be used to estimate standard errors of free-energy differences.

## 3 Implementation

The metadynminer.py library is provided via Python package managers PyPI and Conda under the GPL-3.0 license. The source code is available at GitHub. The program is tested by Continuous Integration service at the latest Ubuntu, MacOS and Microsoft Windows using Python 3.8–3.12. The GitHub repository provides support and the opportunity for other researchers to contribute. Detailed documentation for the metadynminer.py library is available online at https://metadynreporter.cz.

Jupyter Notebook with the latest version of metadynminer.py is also available to users through Binder technology. The user can choose between the service supported by https://e-infra.cz (higher computational resources, registration required) or by https://mybinder.org (lower computational resources, without registration).

## 4 Conclusions and future perspectives

We have created next-generation software for analysis and visualization of results from metadynamics simulations. It is flexible, easy to use, and freely available, and we believe that it will be very beneficial to the molecular simulation community.

There are other methods inspired by metadynamics currently gaining popularity, for example, Variationally Enhanced Sampling (Valsson and Parrinello 2014) or On-the-fly Probability Enhanced Sampling (Invernizzi and Parrinello 2020). Our library is already flexible enough to address many other metadynamics-related methods. However, we will follow recent development in the field and provide support for new methods to provide an easy-to-use tool with high performance.

Another important topic is the accuracy of prediction made by metadynamics, in particular, random errors. We have developed an easy-to-use estimator for the accuracy of the molecular dynamics simulation (Kříž *et al.* 2023) and plan to extend it to metadynamics. Here, we present autocorrelation analysis as an alternative (see Supplementary Data).

Due to open nature of our code and its availability at GitHub, it is open to other user contributions.

## Supplementary Material

btae614_Supplementary_Data
