# Supplementary material for: Analysis of metadynamics simulations by metadynminer.py
Source: Bioinformatics. 2024 Oct 18;40(10):btae614. doi: 10.1093/bioinformatics/btae614 (PMC11512590; doi:10.1093/bioinformatics/btae614)
Supplement: btae614_Supplementary_Data [file btae614_supplementary_data.pdf]

Supplementary Data for Analysis of metadynamics simulations by metadynminer.py

Jan Beránek, Aleš Křenek and Vojtěch Spiwok

Computational details of Oxytocin metadynamics simulation

Molecular dynamics simulation of oxytocin in water with metadynamics was conducted using GROMACS 2018.6 patched with Plumed 2.5.0 (Tribello et al. [2014]). Relevant preparation steps were conducted using GROMACS 2022.3 (Hess et al. [2008]). The structure of oxytocin was obtained from PDB database, code 7RYC (Meyerowitz et al. [2022]). Topology was built with GROMACS with the Amber14sb_OL15 force field (Maier et al. [2015]).

Simulation box was cubic with a size of 5.23405 nm. The molecule was solvated by TIP3P water. One chloride anion were added, so the net charge of the system was neutral. Potential energy of the system was then minimized using the steepest descent method, until the maximum force acting on any atom was lower than 100 kJ/mol/nm. This step was followed by isothermal-isochoric equilibration at 300 K and isothermal-isobaric equilibration at 300 K and 1 bar, each 100 ps long. Equilibration was followed by 1 ns long molecular dynamics simulation of the system.

Then, 800 ns long well-tempered metadynamics simulation was conducted. Two collective variables (CV) were used, one of them named `torsion_SS` was describing the dihedral angle of disulfide bond defined by atoms Cys1-Cβ, Cys1-Sγ, Cys6-Sγ, Cys6-Cβ. The other CV named `torsion_cycle` was defined as dihedral angle between atoms Cys1-Cα, Ile3-N, Gln4-C and Cys6-Cα and was capturing different conformations of the intramolecular ring of the oxytocin molecule. Bias potential was added every 1 ps, initial height of the hill was set to 1.0 kJ/mol, width of hills for both CVs was 0.3 rad. Biasfactor was equal to 10.

In the relevant steps of equilibration and production simulation run, the following parameters were used: Leap-frog integrator, radius for short-range electrostatic and van der Waals was set to 1 nm. Particle Mesh Ewald method (Darden et al. [1993]) was used for computing long-range electrostatic interactions. Temperature coupling was conducted using Parrinello-Bussi thermostat (Bussi et al. [2007]) and pressure coupling was conducted with Parrinello-Rahman barostat (Parrinello and Rahman [1981]). The files required to run this simulation are presented in Plumed Nest (The PLUMED consortium [2019]) (https://www.plumed-nest.org/eggs/24/010/)

Comparison of FESs calculated by Plumed and metadynminer.py

In Figures S1-S6, you can see visualisations of differences between free energy surface (FES) calculated from the same HILLS files by Plumed version 2.8.0 and by metadynminer.py. Figure pairs S1-S2, S3-S4 and S5-S6 were calculated based on 1D, 2D and 3D HILLS files, respectivelly. In Figures S1, S3 and S5, the slower and precise algorithm was invoked by specifying `original=True` and the differences from Plumed's calculation are in the order of tenths of µJ/mol, which corresponds to machine precision level. In Figures S2, S4 and S6, faster algorithm was used for hills summation, its errors are in the order of units of kJ/mol for 1D and 2D FESs and in the order under tens of kJ/mol for 3D FES. This makes the fast algorithm useful for casual visualisation purposes. We have observed that the errors of the fast algorithm tend to be larger if the FES being calculated has more dimensions or lower resolution.

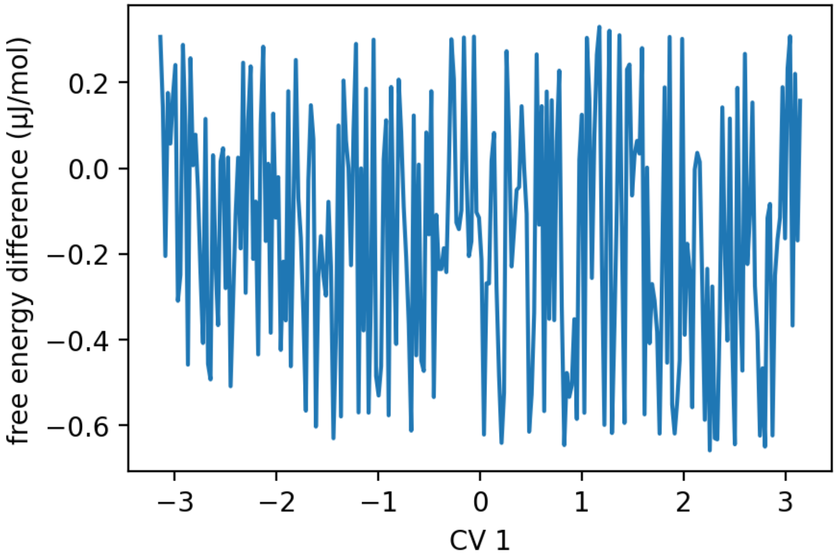

Fig. S1: Visualisation of the difference between one dimensional FES with resolution 256 bins calculated by Plumed 2.8.0 versus calculated by metadynminer.py using `original=True`.

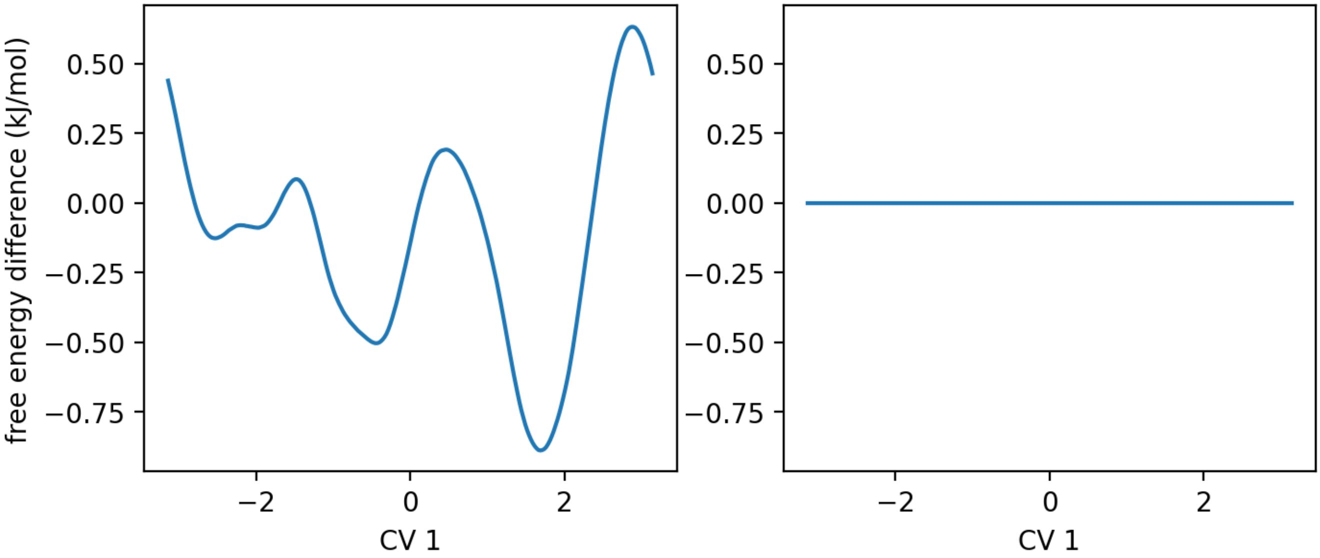

Fig. S2: Visualisation of the difference between one dimensional FES with resolution of 256 bins calculated by Plumed 2.8.0 versus calculated by metadynminer.py using `original=False` (left) compared to the difference between FES calculated by Plumed 2.8.0 versus calculated by metadynminer.py using `original=True` (right).

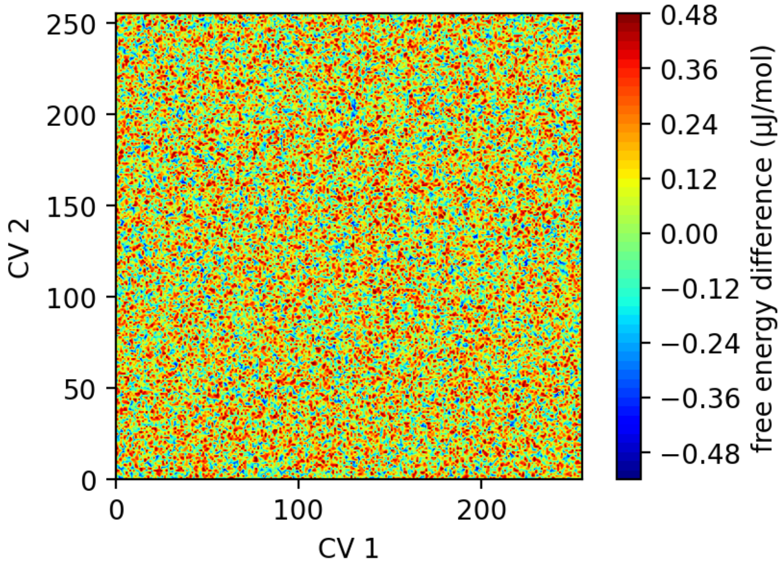

Fig. S3: Visualisation of the difference between two dimensional FES with resolution 256x256 bins calculated by Plumed 2.8.0 versus calculated by metadynminer.py using `original=True`.

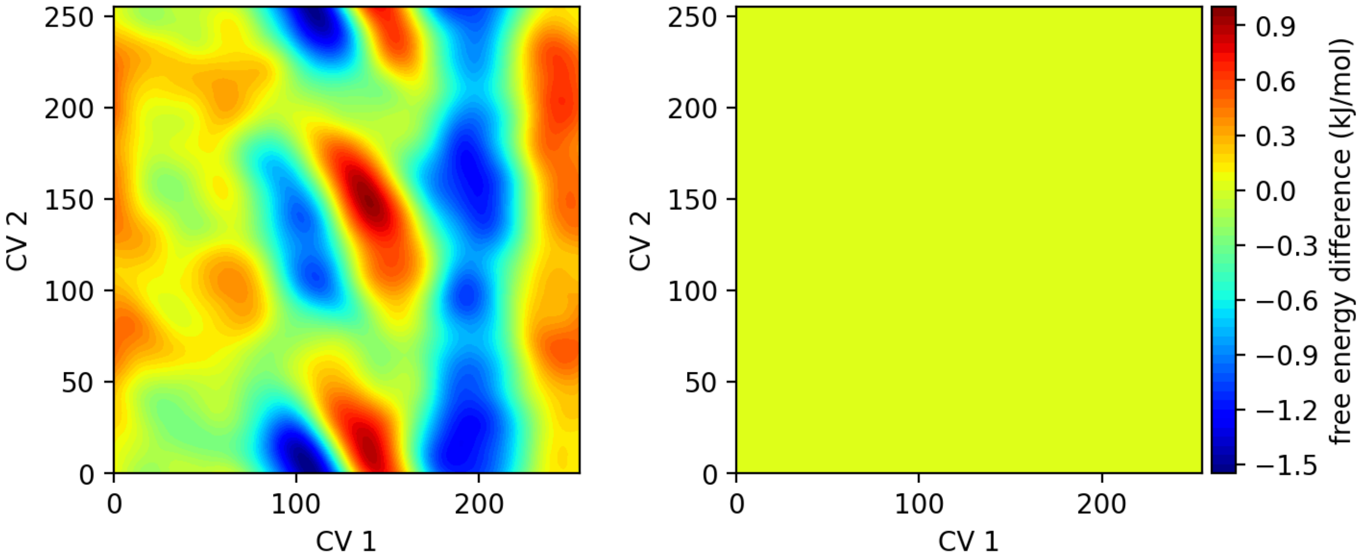

Fig. S4: Visualisation of the difference between two dimensional FES with resolution 256x256 bins calculated by Plumed 2.8.0 versus calculated by metadynminer.py using `original=False` (left) compared to the difference between FES calculated by Plumed 2.8.0 versus calculated by metadynminer.py using `original=True` (right).

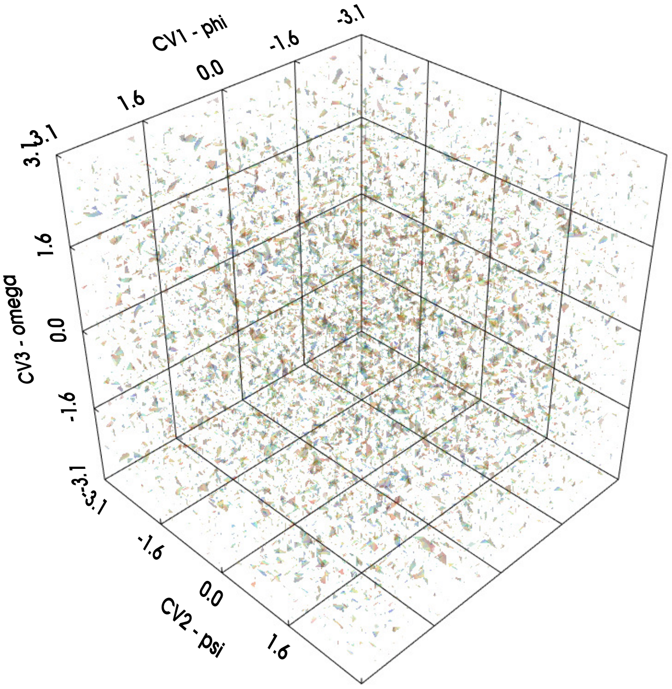

Fig. S5: Visualisation of the difference between three dimensional FES with resolution 64x64x64 bins calculated by Plumed 2.8.0 versus calculated by metadynminer.py using `original=True`. Isosurface with free energy difference = 0 kJ/mol is visualised.

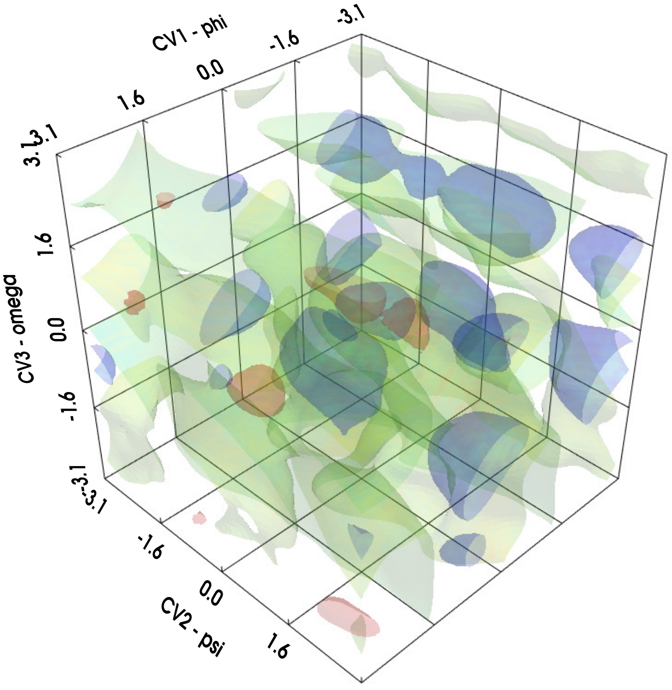

Fig. S6: Visualisation of the difference between three dimensional FES with resolution 64x64x64 bins calculated by Plumed 2.8.0 versus calculated by metadynminer.py using `original=False`. Isosurfaces with free energy difference equal to -5.0 kJ/mol, 0.0 kJ/mol and + 5.0 kJ/mol are visualised in blue, green and red respectively.

Export free energy surface plot from metadynminer.py to a html file

This is an example of python code which can be used to produce interactive html file from matplotlib figure containing free energy surface visualisation made with metadynminer.py.

```
import mpld3
import metadynminer as mm

hills = mm.Hills()
fes = mm.Fes(hills)
fig = fes.plot(contours_spacing=10, return_fig=True)

html = mpld3.fig_to_html(fig)

with open('test.html','w') as file:
    file.write(html)
```

Export free energy surface from metadynminer.py to an stl file

This is an example of python code which can be used to produce 3D printable stl file based on free energy surface made with metadynminer.py.

```
from stl import mesh
import metadynminer as mm

hills = mm.Hills()
fes = mm.Fes(hills)

f3d_mean_size = np.mean([fes.cv1max-fes.cv1min, fes.cv2max-fes.cv2min])
f3d_offset=0.1
fe_range = fes.fes.max()-fes.fes.min()
n = fes.fes.shape[0]

heightmap = (fes.fes*fes.res/2)/fe_range

heightmap += f3d_offset * np.max(heightmap)

# Create an empty list to store the vertices and faces
vertices = []
faces = []

# Generate vertices for the top surface
for i in range(n):
    for j in range(n):
        vertices.append([i, j, heightmap[i, j]])

# Generate vertices for the bottom surface (height = 0)
for i in range(n):
    for j in range(n):
        vertices.append([i, j, 0])

# Generate faces for the top surface
for i in range(n - 1):
    for j in range(n - 1):

        # Triangle 1
        faces.append([
            i * n + j,
            i * n + (j + 1),
            (i + 1) * n + j
        ])
```

```
        # Triangle 2
        faces.append([
            (i + 1) * n + j,
            i * n + (j + 1),
            (i + 1) * n + (j + 1)
        ])

# Generate faces for the sides
for i in range(n - 1):
    # Front side (j=0)
    faces.append([i * n, (i + 1) * n, (i + 1) * n + n * n])
    faces.append([i * n, (i + 1) * n + n * n, i * n + n * n])

    # Back side (j=n-1)
    faces.append([i * n + (n - 1), (i + 1) * n + (n - 1), (i + 1) * n + (n - 1) + n * n])
    faces.append([i * n + (n - 1), (i + 1) * n + (n - 1) + n * n, i * n + (n - 1) + n * n])

for j in range(n - 1):
    # Left side (i=0)
    faces.append([j, j + 1, j + 1 + n * n])
    faces.append([j, j + 1 + n * n, j + n * n])

    # Right side (i=n-1)
    faces.append([(n - 1) * n + j, (n - 1) * n + (j + 1), (n - 1) * n + (j + 1) + n * n])
    faces.append([(n - 1) * n + j, (n - 1) * n + (j + 1) + n * n, (n - 1) * n + j + n * n])

# Generate faces for the bottom surface
for i in range(n - 1):
    for j in range(n - 1):
        base_index = n * n
        # Triangle 1
        faces.append([
            base_index + i * n + j,
            base_index + i * n + (j + 1),
            base_index + (i + 1) * n + j
        ])

        # Triangle 2
        faces.append([
            base_index + (i + 1) * n + j,
            base_index + i * n + (j + 1),
            base_index + (i + 1) * n + (j + 1)
        ])

# Convert to numpy arrays
vertices = np.array(vertices)
faces = np.array(faces)

# Create the mesh
surface_mesh = mesh.Mesh(np.zeros(faces.shape[0], dtype=mesh.Mesh.dtype))

for i, f in enumerate(faces):
    for j in range(3):
        surface_mesh.vectors[i][j] = vertices[f[j], :]

surface_mesh.save("output.stl")
```

Autocorrelation analysis with metadynminer.py

This is an example of code performing autocorrelation analysis based on the data from metadynamics simulation, using metadynminer.py and numpy libraries. Matplotlib.pyplot is used to visualise intermediate results.

```
import numpy as np
import metadynminer_debug as mm
from matplotlib import pyplot as plt

hills = mm.Hills("oxytocin_whole", periodic=[True,True])
fes = mm.Fes(hills)
minima = mm.Minima(fes, precise=True)

# show the list of local minima and visualise FES with minima to decide,
# which pair of minima should have their free energy difference analysed
# further:

minima.minima
minima.plot()

# load the files (COLVAR_n) generated during metadynamics simulation which
# contain information about the CV values and corrected bias potential
# (rbias)

colvar1 = np.loadtxt("COLVAR_1")
colvar2 = np.loadtxt("COLVAR_2")
colvar3 = np.loadtxt("COLVAR_3")
colvar4 = np.loadtxt("COLVAR_4")
colvar5 = np.loadtxt("COLVAR_5")
colvar6 = np.loadtxt("COLVAR_6")
colvar7 = np.loadtxt("COLVAR_7")
colvar8 = np.loadtxt("COLVAR_8")
colvar = np.concatenate((colvar1[1:,:],
                         colvar2[1:,:],
                         colvar3[1:,:],
                         colvar4[1:,:],
                         colvar5[1:,:],
                         colvar6[1:,:],
                         colvar7[1:,:],
                         colvar8[1:,:]))

# calculate the bins on FES where the system was present during
# the simulation:

cv1bins = ((colvar[:,1]-fes.cv1min)*fes.res/(fes.cv1max - fes.cv1min)).astype(np.int64)
cv2bins = ((colvar[:,2]-fes.cv1min)*fes.res/(fes.cv1max - fes.cv1min)).astype(np.int64)

# load corrected bias potential and calculate weight of samples

kT = 300.0*8.314/1000.0

rbias = colvar[:,4]
weight = np.exp(rbias/kT)

# find out, to which local minima the system belongs during the simulation
# save this information to a numpy vektor with values 1 for minimum A,
# 2 for minimum B etc.

minima_list =  minima.m_fes[cv1bins, cv2bins]

# calculate cumulative sum of populations for minima A and B and
# corresponding free energies and their difference

pop1 = np.cumsum((minima_list == 1) * weight)
pop2 = np.cumsum((minima_list == 2) * weight)
pop1[pop1 == 0.] = np.nextafter(0.,1)
```

```
pop2[pop2 == 0.] = np.nextafter(0.,1)
fe1 = -kT*np.log(pop1)
fe2 = -kT*np.log(pop2)
fe2_1 = fe2 - fe1

# visualise the difference between free energies of the two minima
# and decide from which time of the simulation to continue the analysis
# (skip the initial equilibration phase)

plt.plot(fe2_1)
plt.ylim(-10, 10) # adjust the y range as necessary

# for the following calculation use only samples after
# for example the 100.000th sample:

fe2_1 = fe2_1[100000:]

# perform auocorrelation analysis:

nsamples = len(fe2_1)

fe2_1m = np.mean(fe2_1)

deltas = fe2_1 - fe2_1m

denom = np.sum(deltas*deltas)/float(nsamples)

ssauto = 0.0
for k in range(1, nsamples-1):
  sauto = np.sum(deltas[1:nsamples-k]*deltas[-(nsamples-k-1):])/(float(nsamples)-float(k))
  if sauto <= 0.0 and k > 3:
     break
  ssauto = ssauto + 2.0*sauto*(1.0-float(k)/float(nsamples))
ssauto = ssauto / denom + 1.0

nsamples2 = np.floor(nsamples/ssauto)

selected = fe2_1[np.round(np.arange(int(nsamples2)+1)*ssauto).astype(int)]
fe2_1ac = np.mean(selected)


sem = np.std(selected)/np.sqrt(nsamples2)

# print the results:

print(f"Mean free energy difference: {fe2_1ac}, standard error of the mean: {sem}.")
```

References

G. Bussi, D. Donadio, and M. Parrinello. Canonical sampling through velocity rescaling. *J. Chem. Phys.*, 126(1):014101, 2007. doi: 10.1063/1.2408420.

T. Darden, D. York, and L. Pedersen. Particle mesh ewald: An $n \cdot \log(n)$ method for ewald sums in large systems. *J. Chem. Phys.*, 98 (12):10089–10092, 1993. doi: 10.1063/1.464397.

B. Hess, C. Kutzner, D. van der Spoel, and E. Lindahl. Gromacs 4: Algorithms for highly efficient, load-balanced, and scalable molecular simulation. *J. Chem. Theory Comput.*, 4(3):435–447, 2008. doi: 10.1021/ct700301q.

J. A. Maier, C. Martinez, K. Kasavajhala, L. Wickstrom, K. E. Hauser, and C. Simmerling. ff14SB: Improving the accuracy of protein side chain and backbone parameters from ff99SB. *J. Chem. Theory Comput.*, 11(8):3696–3713, 2015. doi: 10.1021/acs.jctc.5b00255.

J. G. Meyerowitz, M. J. Robertson, X. Barros-Álvarez, O. Panova, R. M. Nwokonko, Y. Gao, and G. Skiniotis. The oxytocin signaling complex reveals a molecular switch for cation dependence. *Nat. Struct. Mol. Biol.*, 29:274—281, 2022. doi: 10.1038/s41594-022-00728-4.

M. Parrinello and A. Rahman. Polymorphic transitions in single crystals: A new molecular dynamics method. *J. Appl. Phys.*, 52(12): 7182–7190, 1981. doi: 10.1063/1.328693.

The PLUMED consortium. Promoting transparency and reproducibility in enhanced molecular simulations. *Nat. Methods*, 16:670–673, 2019. doi: 10.1038/s41592-019-0506-8.

G. A. Tribello, M. Bonomi, D. Branduardi, C. Camilloni, and G. Bussi. PLUMED 2: New feathers for an old bird. *Comput. Phys. Com.*, 185(2):604–613, 2014. doi: 10.1016/j.cpc.2013.09.018.
